# Demographic Histories and Genome-Wide Patterns of Divergence in Incipient Species of Shorebirds

**DOI:** 10.3389/fgene.2019.00919

**Published:** 2019-11-08

**Authors:** Xuejing Wang, Kathryn H. Maher, Nan Zhang, Pinjia Que, Chenqing Zheng, Simin Liu, Biao Wang, Qin Huang, De Chen, Xu Yang, Zhengwang Zhang, Tamás Székely, Araxi O. Urrutia, Yang Liu

**Affiliations:** ^1^State Key Laboratory of Biocontrol, Department of Ecology, School of Life Sciences, Sun Yat-sen University, Guangzhou, China; ^2^Milner Centre for Evolution, Department of Biology and Biochemistry, University of Bath, Bath, United Kingdom; ^3^Department of Animal and Plant Sciences, University of Sheffield, Sheffield, United Kingdom; ^4^Ministry of Education Key Laboratory for Biodiversity and Ecological Engineering, College of Life Sciences, Beijing Normal University, Beijing, China; ^5^Department of Bioinformatics, Shenzhen Realomics Biological Technology Ltd, Shenzhen, China; ^6^School of Biosciences, University of Melbourne, Parkville, VIC, Australia; ^7^Instituto de Ecología, Universidad Nacional Autónoma de México, Ciudad de México, Mexico

**Keywords:** speciation, population genomics, shorebirds, gene flow, natural selection

## Abstract

Understanding how incipient species are maintained with gene flow is a fundamental question in evolutionary biology. Whole genome sequencing of multiple individuals holds great potential to illustrate patterns of genomic differentiation as well as the associated evolutionary histories. Kentish (*Charadrius alexandrinus*) and the white-faced (*C. dealbatus*) plovers, which differ in their phenotype, ecology and behavior, are two incipient species and parapatrically distributed in East Asia. Previous studies show evidence of genetic diversification with gene flow between the two plovers. Under this scenario, it is of great importance to explore the patterns of divergence at the genomic level and to determine whether specific regions are involved in reproductive isolation and local adaptation. Here we present the first population genomic analysis of the two incipient species based on the *de novo* Kentish plover reference genome and resequenced populations. We show that the two plover lineages are distinct in both nuclear and mitochondrial genomes. Using model-based coalescence analysis, we found that population sizes of Kentish plover increased whereas white-faced plovers declined during the Last Glaciation Period. Moreover, the two plovers diverged allopatrically, with gene flow occurring after secondary contact. This has resulted in low levels of genome-wide differentiation, although we found evidence of a few highly differentiated genomic regions in both the autosomes and the Z-chromosome. This study illustrates that incipient shorebird species with gene flow after secondary contact can exhibit discrete divergence at specific genomic regions and provides basis to further exploration on the genetic basis of relevant phenotypic traits.

## Introduction

Understanding the conditions in which speciation occurs is a fundamental question in evolutionary biology. Of equal importance is the understanding of how newly diverged species (incipient species) are maintained, as it is likely that interspecific gene flow is a common occurrence between diverging species (Payseur and Rieseberg, 2016). During allopatric speciation, a physical barrier acts to prevent gene flow across the whole genome (Via, 2012) and pre- and post-zygotic mechanisms of reproductive isolation can evolve to facilitate divergence. However, gene flow across geographical barriers, or secondary contact between diverged populations is possible, which allows gene flow to recommence (Wu, 2001). Even infrequent gene flow can erode species barriers (Lindtke and Buerkle, 2015). In the more contentious geographical context, such as parapatric or sympatric speciation (Bird et al., 2012; Bolnick and Fitzpatrick, 2007), disentangling the relative role of gene flow and other diverging conditions and forces remains challenging (Shaner et al., 2015; Wang et al., 2017).

Whether speciation can occur with gene flow has been an area of intense investigation within the last decade (Wolf and Ellegren, 2017). In certain instances, gene flow overcomes the barriers of reproductive isolation and reverse speciation processes occur, while in other species divergence persists in spite of gene flow (Seehausen, 2006; Taylor et al., 2006; Webb et al., 2011; Feder et al., 2012; Martin et al., 2013). The homogenizing effect of gene flow, on both a genetic and phenotypic level, can be reduced in sympatry and at secondary contact areas if the diverging species vary in niche or mate preference (Nosil and Sandoval, 2008; Merrill et al., 2011; McLean and Stuart-Fox, 2014; Poelstra et al., 2014; Shaner et al., 2015). Areas of elevated genetic differentiation found throughout the genome, so called ‘genomic islands’, or a subset of these regions, could be responsible for the phenotypic differences observed between species or contain potential mechanisms for reproductive isolation and hybrid incompatibility (Turner et al., 2005; Harr, 2006; Nosil et al., 2009; Ellegren et al., 2012; Poelstra et al., 2014). These regions of high genetic differentiation are often widespread throughout the genome and can be either small (e.g. Nadeau et al., 2012) or large in size (e.g. Renaut et al., 2012). As gene flow leaves detectable signatures of divergence in the introgressed regions of genome (Payseur and Rieseberg, 2016), it is possible to study these patterns and infer past gene flow and also the demographic history of a species (Roux et al., 2013; Malinsky et al., 2015; Payseur and Rieseberg, 2016).

Improved estimation of demographic history makes it possible to better understand population differentiation and speciation mechanisms (Nadachowska-Brzyska et al., 2013). It allows estimates of gene flow, divergence time, effective population size (measured in number of diploid individuals) and population changes in size throughout time. It is also possible to obtain information on which is the most likely demographic history of speciation, such as isolation, isolation with migration, early migration or secondary contact. Such inference of demographic history could also help identify species and populations at risk of extinction (Allendorf et al., 2010; Li et al., 2014; Xue et al., 2016), or populations with unique genetic structure worthy of conservation effort (i.e. Zhao et al., 2013; Foote et al., 2019). Using whole-genome approaches also makes it possible to screen for fast evolving regions along genomes that techniques using a small number of genetic markers may miss (Toews et al., 2016). For example, genomic resequencing in carrion (*Corvus corone*) and hooded crows (*C. cornix*) found that distinct differences in phenotype are maintained by variation in less than 1% of the genome (Poelstra et al., 2014). Assessing how secondary contact and hybridization between distinct taxa impacts native populations is of vital importance when considering how to implement effective conservation protocols (Allendorf et al., 2001; Fitzpatrick et al., 2015). Advancements in analysis on the basis of coalescence simulations (Beaumont, 2010; Arenas, 2015) using high-throughput genomic data, hold great potential in making inferences about demographic histories (Ellegren, 2014).

*Charadrius* plovers are model species for investigating breeding system evolution and have been used in numerous studies to better understand mating and parenting behaviors (Kosztolányi et al., 2006; Kosztolányi et al., 2009; Székely, 2014; Bulla et al., 2016; Eberhart-Phillips et al., 2017; Maher et al., 2017). Species in the Kentish plover complex (*Charadrius alexandrinus*; KP) are small shorebirds found breeding on saline lakes and coasts throughout Eurasia and North Africa (del Hoyo et al., 1996). A previous study found no genetic differentiation between several Eurasian continental populations of Kentish plover (Küpper et al., 2012). In East Asia, the subspecies *C. a. alexandrinus* has a wide breeding range in the temperate zone, whereas the southern subspecies *C. a. dealbatus*, (known as white-faced plovers; WFP) show distinct phenotypic traits compared to northern populations. They lack the dark eye barring of the KP, and have lighter lower ear coverts, a brighter cinnamon cap and paler plumage with white lores (Rheindt et al., 2011). They also typically have longer wings, beak and tarsus and are more commonly found on sandier substrates, with more active foraging behavior and a more upright stance (Rheindt et al., 2011). While the KP and WFP share much of the same wintering range, they have largely non-overlapping breeding ranges. WFPs breed exclusively in a restricted coastal range from Fujian to Guangxi, as well as Hainan Island in south-east China. KPs nest to the north of this range (Wang et al., 2019). Previous work examining mitochondrial DNA and microsatellite markers has shown that although KPs and WFPs are phenotypically well-differentiated (Kennerley et al., 2008; Rheindt et al., 2011), genetically, they lack differentiation. More extensive microsatellite genotyping and autosomal nuclear sequencing (Wang et al., 2019) as well as genome-wide SNP identification and genotyping (Sadanandan et al., 2019), however, showed KPs and WFPs are distinct and young lineages. The two lineages diverged around 0.6 Ma with evidence of bidirectional gene flow (Wang et al., 2019).

Here, we expand on previous works by characterizing demographic histories and genomic landscape of divergence in two closely related plovers. The isolation-with-migration model (IM) applied in a previous study assumes gene flow throughout the entire divergence history of the two plover lineages (Wang et al., 2019) but it is not known whether gene flow persisted in the early stages of divergence or/and also occurred after the secondary contact. The current work explores these different scenarios of gene flow based on advanced modelling on historical demography. Further, under the model of speciation-with-gene-flow model (Seehausen, 2006; Taylor et al., 2006; Webb et al., 2011; Feder et al., 2012; Martin et al., 2013), it is relevant to investigate the potential heterogeneous genomic landscape of incipient species. In the case of the two study species, it is possible that a small number of genomic regions are involved in the phenotypic and ecological differences between them (Rheindt et al., 2011; Toews et al., 2016). Hence, we attempted to disentangle the aforementioned questions by applying whole-genome sequencing and assembly of a high-quality *de novo* reference genome of a female KP. We also re-sequenced whole genomes of 21 unrelated male genomes from five populations of KP and six populations of WFP in China as well as full mitochondrial genomes of four KP and two WFP.

## Materials and Methods

### Sampling Collection

**Figure 1:**
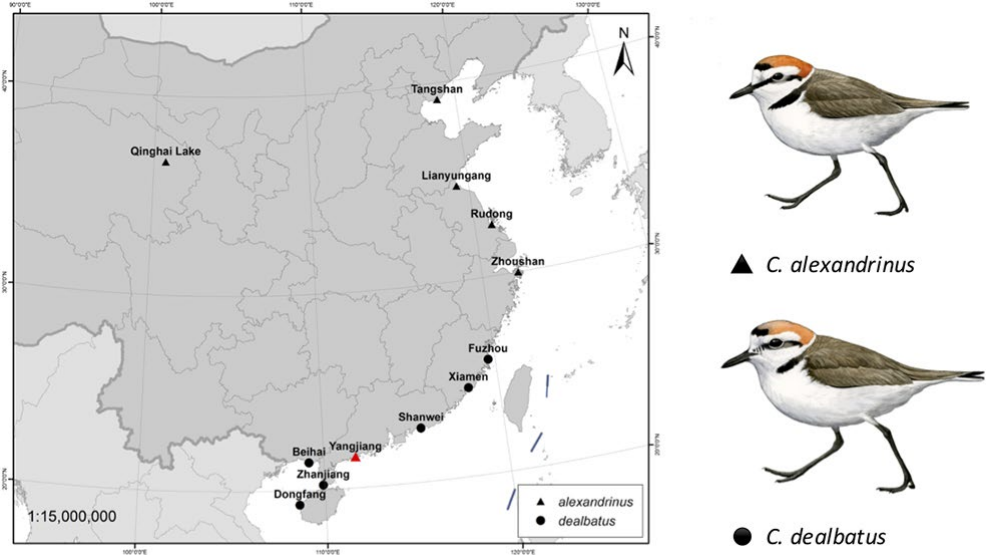
Sampling locations of two plover species, Kentish plover *Charadrius alexandrinus* and white-faced plover *C. dealbatus*. The red triangle represents the location where one individual of Kentish plover for was collected for *de novo* sequencing.

A single female KP (heterogametic sex) was collected using mist nets in coastal Xitou, Yangjiang county, Guangdong, China in November 2014. A muscle sample was taken from this individual, stored in RNALater (QIAGEN, USA) and transported to the sequencing center for *de novo* whole genome sequencing (BGI-Shenzhen). In addition, twenty male KP and WFP were collected from breeding colonies at 11 sites for whole genome resequencing (**Figure 1** and **Supplementary Table 1**), including one inland site at Qinghai Lake, and one continental island, Hainan, with the remaining sites located along the Chinese coast, starting from Hebei to Guangxi. Using males prevents systematic biases caused by differences in coverage of the autosomes and Z chromosome from occurring (Poelstra et al., 2014). One female WFP was collected from Hainan for resequencing in higher coverage. These individuals were captured on nests using funnel traps during the breeding season between March and July in 2014–2015 (Székely et al., 2011). Blood samples taken from these individuals were stored in RNALater (QIAGEN, USA) at −40°C. This study did not involve endangered or protected species. All the bird captures and sampling were performed with permission from the respective authorities (Beijing Normal University to PQ and Sun Yat-sen University to YL) and blood and tissue collection procedures conform to the regulations of the animal experimental and medical ethics committee of Sun Yat-sen University. This study was carried out in accordance with the principles of the Basel Declaration and recommendations of the Institutional Ethical Committee of Animal Experimentation of Sun Yat-sen University (2005DKA21403-JK).

### *De Novo* Sequencing and Assembly of Kentish Plover Genome

We isolated DNA from blood/muscle samples using Qiagen DNeasy Blood and Tissue Kit using standard manufacturer protocols. Short-insert-sized (170 and 800bp) and mate-pair (2, 5, 10, and 20kb) DNA libraries were constructed for the KP reference genome (**Supplementary Table 2**). All libraries were sequenced using Illumina Hiseq 2000. Genomic DNA paired-end sequence data were assembled using short oligonucleotide analysis package SOAPdenovo (Luo et al., 2012). Final N50 contig and scaffold sizes were calculated based on a minimum length of sequence >100bp. The sequencing coverage, depth, and GC content distribution were evaluated by mapping all sequencing reads of the short-insert-sized libraries back to the scaffolds using BWA (Li and Durbin, 2009) with the algorithm of BWA-MEM. We also evaluated the genome assembly completeness using BUSCO’s genome mode (Simão et al., 2015). After the assembly, we carried out genome annotation and characterization (see the **Supplementary Materials 3.1–3.4**).

### Whole-Genome Resequencing

We extracted genomic DNA from blood samples of 20 male individuals of KP and WFP, 10 of each species for 5x depth resequencing and one female WFP from Dongfang, Hainan for 30x depth. For each individual, 1–3 μg of DNA was sheared into fragments of 200–800 bp with the Covaris system. DNA fragments were then treated according to the Illumina DNA sample preparation protocol: fragments were end repaired, A-tailed, ligated to paired-end adaptors, and PCR amplified with 500-bp inserts for library construction. Sequencing was performed on the Illumina HiSeq 2000 platform, and 100-bp paired-end reads were generated. To avoid reads causing artificial bias during the library construction and sequencing process (i.e. low quality reads, which mainly resulted from base-calling duplicates and adapter contamination), we carried out quality control and filtered out sequences using FastQC (Andrews, 2010), Duplication150 (https://github.com/Holt59/cakephp3-bootstrap-helpers/pull/150) and Trimmomatic (Bolger et al., 2014) according to the following criteria: (a) any reads with adapter sequence, allowing ≤10% mismatches; (b) any reads with > 50% bases having phred quality <5; (c) any reads with ≥10% unidentified nucleotides (N).

### Read Mapping and SNP Calling

After quality control, the reads were mapped to the KP genome using BWA and reads having a mean of approximately 5x depth for each individual and >90% coverage of the KP genome were retained for SNP calling. We used GATK v 3.5 (DePristo et al., 2011) program to call SNPs. SNPs were filtered using VCFtools (Danecek et al., 2011) and GATK by following criteria: 1) missing rate <=0.10; 2) allele frequency >0.05; 3) each 10 bp <=3 SNPs.

### Mitochondrion Genome Analysis

To infer evolutionary history from mitochondrial DNA (mtDNA), we conducted mitochondrial genome sequencing. Six blood samples were selected, including four KP from four sites (1. Xinbei, Taiwan; 2. Weihai, Shandong; 3. Qinghai Lake, Qinghai; 4. Zhoushan, Zhejiang) and two WFP from two sites (5. Dongfang, Hainan; 6. Minjiang Estuary Fuzhou, Fujian). Gross genomic DNA was extracted by TIANamp Blood Genomic DNA Extraction Kit (TIANGEN, China), following the standard extraction protocol. Paired-end (PE) 150-bp sequence reads were obtained from Illumina MiSeq PE150 sequencing for each sample. Novogene Ltd. (Beijing) performed the library preparation and sequencing. Consequently, we obtained 31,282,372, 29,185,701, 25,092,010, and 24,383,015 clean paired-end reads for the four KP from four sites, respectively; and 30,397,339 and 24,982,573 clean paired-end reads for the two WFP from two sites, respectively. We mapped the clean reads to the mitochondrial genome of the pied avocet, *Recurvirostra avosetta* (GenBank Accession Number: KP757766), using “Map to Reference” tool in Geneious R8 (Biomatters, Auckland, New Zealand) with a medium-low sensitivity and ran 5 iterations. Consensus sequences were saved using a 75% masking threshold, and sites that received insufficient coverage (<5x) were coded using the IUPAC ambiguity symbol N.

We inferred mitochondrial phylogenetic relationship between the two plovers with Bayesian Inference in MrBayes v.3.2.6 (Ronquist et al., 2012) and maximum likelihood in RAxML v8 (Stamatakis, 2014) using complete mitochondrial genome sequences including the pied avocet as outgroup. MrBayes was run on the CIPRES science Gateway portal (Miller et al., 2010) with Metropolis coupling (four chains) set for 10 million generations and sampling every 10,000 generations, using HKY nucleotide substitution model which was best-fit model tested by jModelTest 2 (Darriba et al., 2012). Tracer v1.6 (Bouckaert et al., 2014) was used to check the effective sample sizes (ESS) for parameter estimation. RAxML was also run on CIPRES with GTRCAT model and 1,000 bootstrap runs. Maximum-likelihood-bootstrap proportions (MLBS) ≥70% were considered strong support (Hillis and Bull, 1993). The phylogenetic trees were visualized using FigTree v1.4.3 (available at: http://tree.bio.ed.ac.uk/software/figtree). 

### Population Structure and Divergent Histories Between the Two Plover Species

To infer population structuring between KP and WFP, we carried out genetic admixture analysis of the resequenced individuals with ADMIXTURE 1.3 (Alexander et al., 2009). For K from 1 to 5, each analysis was performed using 200 bootstraps. We applied two approaches to reconstruct the demographic history of KP and WFP. First, each individual of the two species with high sequencing depth was used to perform a pairwise sequentially Markovian coalescent (PSMC) model to examine changes in historical effective population sizes (*N* *_e_*) of both species (Li and Durbin, 2011). This enabled us to infer demographic dynamics between 10Ka to 10 Ma. The parameters were set as: “N30–t5–r5–p 4 + 30*2+4+6+10”, following Nadachowska-Brzyska et al. (2015). Generation time was set to 2.5 years. The mutation rate was estimated using the method described in Nadachowska-Brzyska et al. (2015) based on branch-specific estimates of the synonymous substitution rate per synonymous site from our dated phylogenomic tree of 16 core waterbirds (see **Supplementary Materials** for further details). One hundred bootstraps were performed for each analysis.

**Figure 2:**
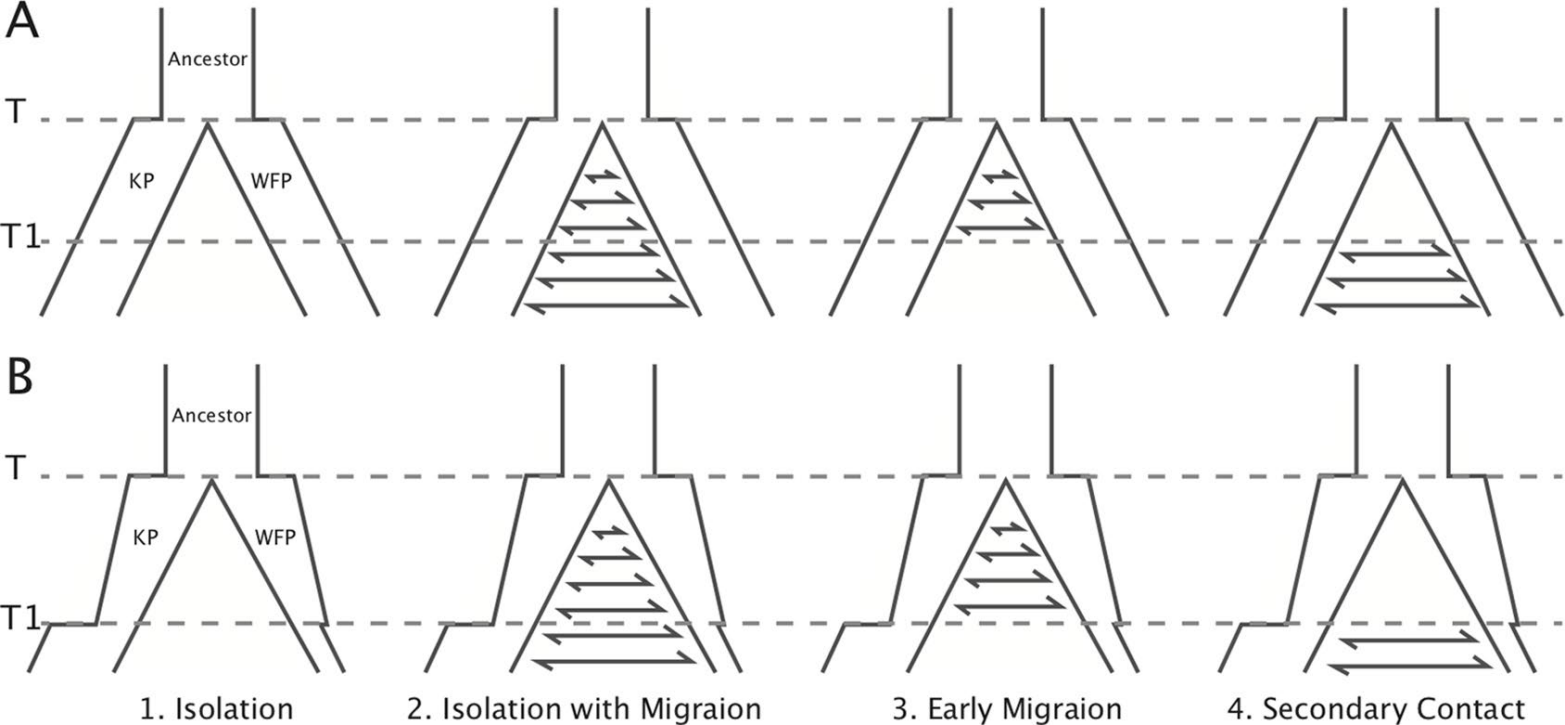
Illustration of the models simulated in ABC analysis. Eight models in two groups were simulated. Effective population sizes in group **(A)** were constant. Effective population sizes in group **(B)** were based on *N* *_e_* changes in PSMC. The PSMC results suggest that the divergence time did not occur earlier than the beginning of the population declines 1 million years ago. To simplify the models, population size shifts and changes of gene flow were set to the same time point (*T1*). Prior ranges are available in **Supplementary Table 17**.

In addition, we carried out a model-based method using an Approximate Bayesian Computational (ABC) approach (Beaumont, 2010) to infer the divergence history between the two plovers using 20 resequenced individuals. To achieve this, we first defined four basic demographic models, including: 1) Isolation model, no gene flow during divergence; 2) Isolation with migration model, constant gene flow during divergence; 3) Early migration model, gene flow only exists within the early period of divergence; 4) Secondary contact model, gene flow only exists in the late period of divergence. We performed two groups of simulations with different effective population size (*N* *_e_*) settings (**Figure 2**). In the first group (A), effective population sizes were hypothesized to be constant in all the four models. In the second group (B), effective population sizes changed based on PSMC results. Model illustrations and priors are shown in **Figure 2** and **Supplementary Table 16**. We used msABC (Pavlidis et al., 2010) to perform coalescent simulations for these eight models. The priors of effective population sizes were set to log-uniform distribution (as suggested in Parag and Pybus, 2019), *T1* was set to normal distribution and other priors as uniform distribution (see **Supplementary Table 16**). The dataset including 20 individuals with 5x sequencing each depth were used to obtain the observed data and priors for simulation. The 10 kbp length loci were randomly chosen from scaffolds with coverage over 80%. The distance between each two loci was higher than 500 Kb to reduce the effect of linkage (Nadachowska-Brzyska et al., 2013). Loci with missing data of over 40% in any individual, or over 30% on average, were excluded (30.3% left). Then *F* *_ST_*, *Tajima’s D* (Tajima, 1989), and LD *r* *^2^* were calculated for each locus with VCFtools 0.1.14. Loci with *F* *_ST_* higher than 0.159 or *Tajima’s D* >1 or <0 were excluded (99.8% left). One hundred forty-three 10-kbp loci with 14,087 SNPs were left for analysis. One million simulations were executed for each model. R package “abc” (Csilléry et al., 2012) was used to choose the model which best fitted the observed data with a tolerance of 0.005. Model selection was performed using a multinomial logistic regression method, first in group A and B separately, and then between groups by using two models with highest likelihood in each group. Five million simulations were used for the best fitted model to estimate population parameters. We used the neural network method with the Epanechnikov kernel to calculate the posterior densities (Blum and François, 2010). The number of neural networks was 50 and tolerance was 0.002. Simulations with *F* *_ST_* higher than 0.159 or *Tajima’s D* >1 or <0 were discarded. All parameters were log-transformed, and medians were used as point estimates.

### Detecting and Annotating Genomic Region Under Selection

In order to better understand the divergence patterns between the two plover species, genetic parameters π, *Tajima’s D*, *F* *_ST_*, and the genome-wide absolute differentiation, denoted as *d* *_f_* (fixed differences) were calculated in 50 kb blocks with VCFtools 0.1.14. The genome-wide absolute difference, *d*_XY_ (Nei, 1987) was calculated with R package “HIERFSTAT” (Goudet, 2005). Blocks with a length shorter than 25kbp were excluded.

In order to map the KP genome scaffolds onto chromosome coordinates, we downloaded the chicken (*Gallus gallus*) genome from NCBI database (GCF_000002315.4) and computed its alignment with the KP reference genome using Satsuma version 3.1.0 (Grabherr et al., 2010). We divided the genome into non-overlapping windows of 50kb in size with the first window of each scaffold beginning with position 1 of that scaffold, oriented along the chromosome. For each window, we estimated the population genomic statistics calculated above. Finally, we generated the genomic landscape of population divergence in KP and WFP according to the above methods.

To calculate *Tajima’s D* per gene we used the R package PopGenome v2.2.3 (Pfeifer et al., 2014) in R v3.3.2 (R Core Team, 2016) for KP and WFP separately. VCF and complementary GFF files were loaded into PopGenome by scaffold with positions with unknown nucleotides excluded (include.unknown = FALSE). Data was then split into genes and neutrality statistics were calculated for coding regions. For calculating *F* *_ST_*, samples were assigned to their two respective species, KP and WFP. *F* *_ST_* statistics were generated per gene for coding regions.

### Gene Ontology Annotations

To obtain gene ontology (GO) categories, Kentish plover proteins were BLASTed against the RefSeq protein database using BLASTP v.3.2.0+, with an *E*-value of 1e-5 (Altschul et al., 1990). GO terms were then assigned using Blast2GO software v.4.1.9 (Götz et al., 2008) and merged with GO terms obtained from InterProScan v.5.25 (parameters: -f xml, -goterms, -iprlookup) (Jones et al., 2014). GO categories were then split into groups associated with biological processes and cellular components. GO categories with fewer than 50 genes were grouped into a single “small” category. Genes with no GO annotation were assigned to an “uncharacterized” category.

### Gene Ontology Enrichment Analyses

Genes were assigned to either autosomes or the Z chromosome, with genes of unknown location excluded from GO enrichment analysis. GO enrichment was performed for genes with high *F* *_ST_*. Genes were considered to have high *F* *_ST_* if they fell above the 95^th^ percentile of *F* *_ST_* values, using R quantile[c(.95)]. Two cut-off values were used, 0.184 for autosomal genes and 0.262 for genes on the Z chromosome. GO enrichment was also performed on genes with high and low *Tajima’s D* values calculated for KP and WFP separately. Genes with positive *Tajima’s D* could indicate balancing selection. Regions of negative *Tajima’s D* can indicate strong positive selection or selective sweeps. Genes were separated into known autosomal and Z chromosomal genes for positive and negative *Tajima’s D* separately. The 95^th^ percentile of positive values were taken as high *Tajima’s D* genes and the 5^th^ percentile were taken for the negative values. This resulted in cut-off values of ≥1.565 for high and ≤−1.179 for low value autosomal genes for KP and ≥1.669 and ≤−1.191 for WFP. ≥1.723 and ≤−1.234 cut-offs were used for the Z chromosome for KP and ≥1.835 and ≤−1.445 for WFP. GO enrichment was performed for both high and low *Tajima’s D* values separately. GO enrichment analyses were performed to evaluate if any GO category was over represented in the set of genes of interest compared to equally sized samples of genes drawn randomly. The expected number of genes annotated to each GO was calculated using 1,000 equally sized random families. Significance was established using Z-scores and a Benjamini-Hochberg correction was applied to adjust for multiple comparisons. GO categories were significantly enriched if the adjusted p value was <0.05. Results were filtered to remove any category with an expected number of genes per category of <1 and an observed number of GOs of 1. This analysis was performed using R v.3.3.2.

## Results

### *De Novo* Sequencing the Kentish Plover Genome

Muscle samples from a heterogametic sex female Kentish plover were collected from a wintering population in Guangdong, China (**Figure 1**). Short read DNA sequencing (125bp) was carried out using the Illumina platform (see **Supplementary Figure 1** for pipeline). After filtering out low quality and clonally duplicated reads, we obtained a genome assembly from 1.81 billion reads in six paired-end and mate-pair libraries that provide 134-fold coverage with a total assembly length of 1.16Gb (**Supplementary Table 2**). This approximates the genome size estimated using K-mer frequency method (**Supplementary Table 3** and **Supplementary Figure 2**). The GC content versus depth is gathered into a cluster showing the genome sequence is pure and has no pollution from other species (**Supplementary Figure 3**).

The contig and scaffold N50 sizes are 38.9 and 3,220.7kbp, respectively, with the largest scaffold spanning 15,291.1kbp (**Supplementary Table 4**). Although the number of scaffolds for the Kentish plover is considerably higher than that of the chicken or the zebra finch genomes, the estimated genome size for the Kentish plover (1,245,524,081bp, ∼1.25 Gb) is comparable to the sequenced genomes of these two species (**Supplementary Table 5**). Whole genome alignment reveals that, as expected, a higher proportion of the zebra finch and KP genomes can be aligned against each other than either of them can to the more distantly related chicken with over 900Mbp that can be aligned between the two species (**Supplementary Table 6**). The BUSCO assessment results indicate that the KP genome assembly has high completeness (C: 94%) (**Supplementary Figure 4**).

### Genome Resequencing Reveals Two Diverging Plover Species With Contrasting Evolutionary Histories

**Figure 3:**
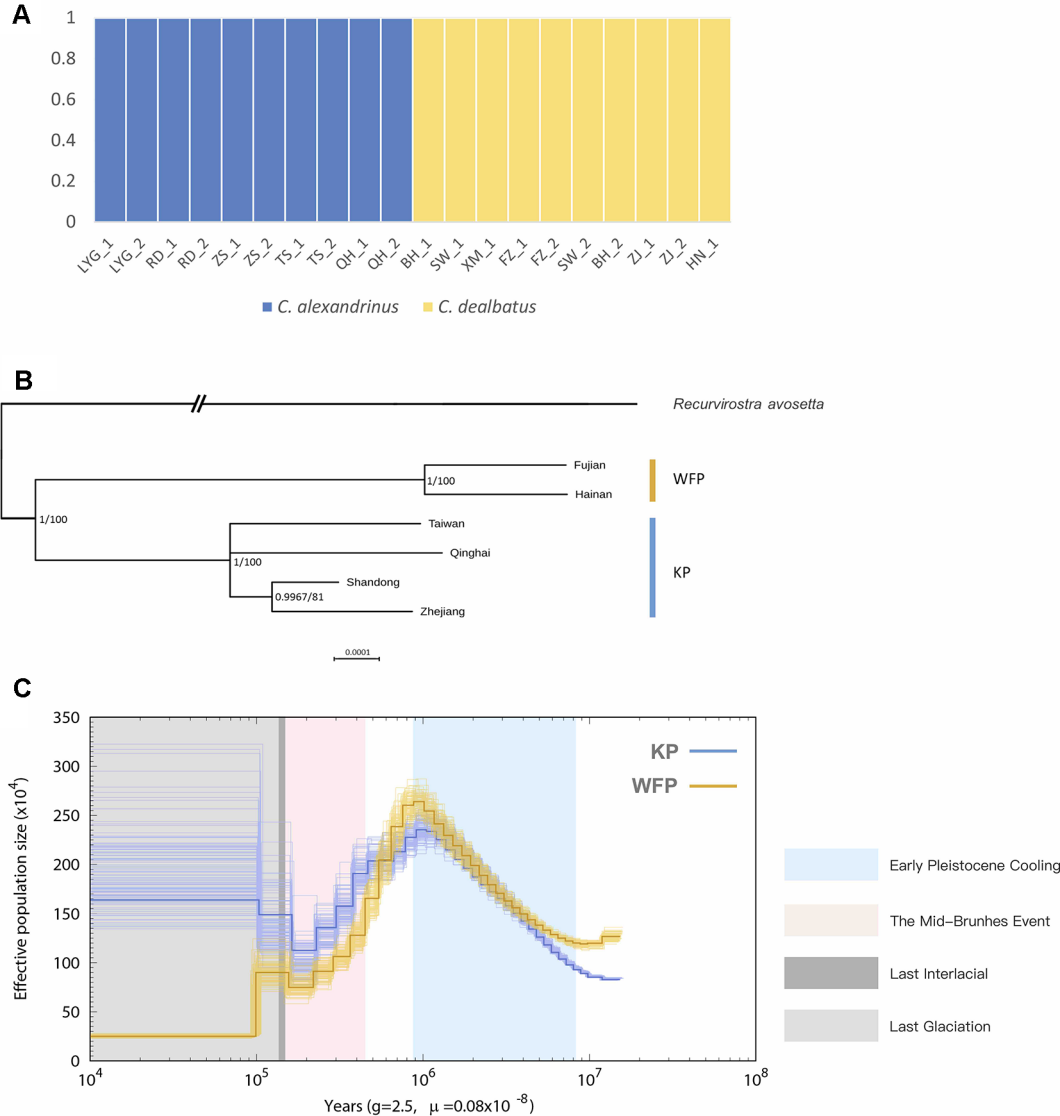
Population genetic structure and historical demography. *C. alexandrinus* marked in blue and *C. dealbatus* in yellow. **(A)** Genetic clustering inferred with ADMIXTURE when K = 2. **(B)** Phylogenetic relationship between the *C. dealbatus* (WFP) and different populations of *C. alexandrinus* (KP) using Bayesian and Maximum Likelihood methods based on mitochondrial genome sequences (c.a. 15kb). Posterior probabilities (pp) and bootstrap supports are indicated at each node. White-faced plover and Kentish plover form two independent evolutionary lineages. **(C)** Demographic history of the Kentish plover, blue line, and white-faced plover, yellow line reconstructed from the reference and population resequencing genomes. The line represents the estimated effective population size (*N* *_e_*), and the 100 thin blue curves represent the PSMC estimates for 100 sequences randomly resampled from the original sequence. Generation time (*g*) = 2.5 years, and neutral mutation rate per generation (μ) = 0.8 × 10^−8^. The Last Interglacial period (LIG, from approx. 130 to 116 ka) is marked by a grey block.

Blood samples were obtained from a total of 21 individuals taken from mainland and Hainan island plover populations along the Chinese coast and from the inland and a high-altitude population of Qinghai Lake (**Figure 1** and **Supplementary Table 1**). Kentish and white-faced plovers have been shown to present distinct phenotypic features, including facial plumage pattern (**Figure 1**) (Kennerley et al., 2008). For the 20 low depth resequenced samples, a total of 914,529,390 high quality paired-end reads were retained for the further analyses (**Supplementary Table 14**). Genome resequencing was carried out resulting in over 95% genome coverage with a depth of over 4x for around 70% of the genome per individual (**Supplementary Table 15**). After filtering, a total of 11,959,725 high quality SNPs were retained. KP and WFP were found to cluster into two distinct groups based on Admixture analysis (**Figure 3A**). For the high depth sequencing WFP, its genome assembly quality was high enough to be used for the PSMC analysis.

In addition, we obtained 15,613 bp of the complete mitochondrial genome for Kentish and white-faced plovers, except for the D-loop region, which had poor assembly quality. In the phylogenetic analysis, topologies between Bayesian and ML tree were consistent (**Figure 3B**). Analyses clearly show that the monophyletic relationship of KP and WFP is strongly supported.

With mutation rate estimated as 8.11 × 10^−8^ per base pair per year, demographic history reconstruction of KP and WFP revealed distinct evolutionary histories of the two species from approximately 10 Ma to 10 ka (**Figure 3C**). PSMC analysis demonstrated a similar *N* *_e_* history for both species around 1 million years ago during the Pleistocene epoch, with population sizes of both species rising from 0.8 to 1 million years ago then sharply declining until about 100 thousand years ago during the Last Interglacial Period. KP and WFP then went through steady population size changes separately. The *N* *_e_* of KP increased greatly to 1.65 million and the *N* *_e_* of WFP decreased to about two hundred and fifty thousand.

With ABC simulations, we found that the genome-wide polymorphism patterns in KP and WFP fit best with the secondary contact model (posterior probability = 0.95, **Supplementary Table 17**), suggesting that the two plover species experienced gene flow after secondary contact. Model selection showed that changing *N* *_e_* models incorporating the PSMC-inferred *N* *_e_* fluctuations had much higher posterior possibilities than constant *N* *_e_* models (Bayes factors > 10^3^), which indicates that the secondary contact model based on PSMC results fitted best (**Figure 2**).

Demographic analyses allowed us to estimate several demographic parameter estimates, including divergence times, effective population sizes (number of diploid individuals) and migration rate per generation (**Table 1**). The two plover populations are estimated to have diverged approximately 863 thousand years ago (95% CI, 844–909 thousand years). The effective population size of KP is estimated to be around 5.6 times higher than that of WFP (median *N* *_e_*_K_ = 3.58 million, 95% CI 3.06–4.66 million, and median *N* *_e_*_W_ = 0.64 million, 95% CI 0.62–1.14 million). The most recent common ancestor is believed to have an effective population size between the two modern species. The estimated gene flow between the two groups of plovers was unequal: c. 236.50 (95% CI 213.64–240.42) individuals per generation immigrate from WFP to KP, and c. 51.48 (95% CI 48.83–57.94) individuals per generation immigrating from KP to WFP (See **Supplementary Figure 10** for all posterior distributions).

**Table 1:** Posterior median, mean, mode, and range of 95% highest probability distribution (HPD) of demographic parameters.

	*T*/10^6^	*N* *_e_*_K_/10^6^	*N* *_e_*_W_/10^6^	*N* *_e_*_A_/10^6^	*2NM*_W- > K_	*2NM*_K- > W_	*T1*/10^6^	*N0*_K_/10^6^	*N0*_W_/10^6^	*G*_K_	*G*_W_
Median	0.863	3.581	0.649	0.752	236.503	51.476	0.059	0.086	0.082	8.090	19.631
Mean	0.875	3.981	0.759	0.739	233.704	51.444	0.057	0.088	0.084	8.087	19.626
Mode	0.861	4.590	0.642	0.811	236.203	51.291	0.059	0.098	0.082	8.089	19.632
2.5%	0.844	3.066	0.622	0.584	213.639	48.829	0.049	0.068	0.076	8.066	19.599
97.5%	0.909	4.656	1.143	0.814	240.422	57.939	0.062	0.110	0.101	8.122	19.644

K represents Kentish plover Charadrius alexandrinus and W represents White-faced plover C. dealbatus. A represents Ancestral population. N_e_: recent effective population size; T: population split time; 2NM: migration rate per generation; T1: time point of changes of population size and migration rate; N0: population size at T1; G: growth rate.

### Genomic Regions Associated With Divergence Between Kentish Plover and White-Faced Plover

**Figure 4:**
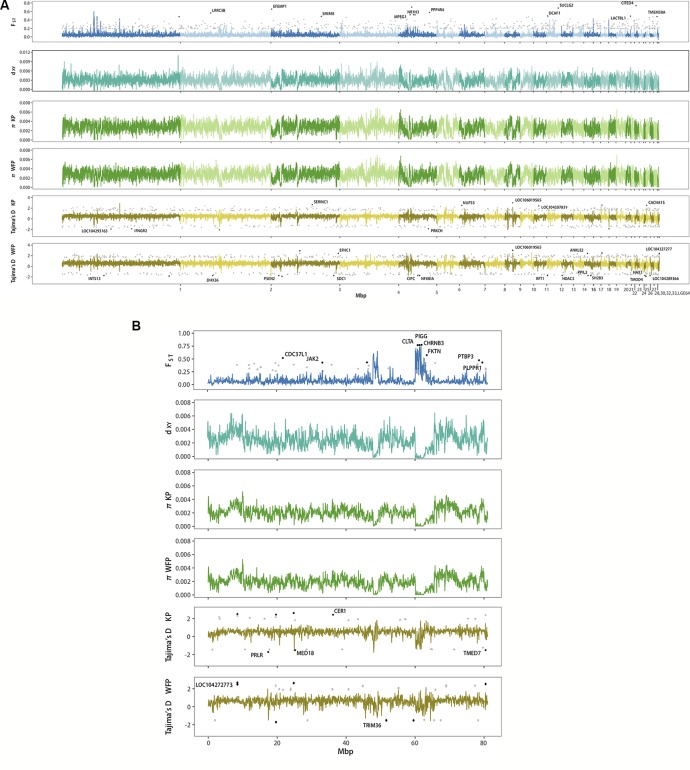
Genome wide landscape of *F* *_ST_*, *d*_XY_, *π*, and *Tajima’s D* for 50 kb sliding window. Different autosomes are marked with alternating light and dark colors. **(A)** Genome wide landscape for autosomes. Ninety-fifth percentile outliers are plotted for *F* *_ST_* and 95^th^/5^th^ percentile outliers are plotted for *Tajima’s D* in grey, as calculated per gene. Ninety-ninth percentile outliers for *F* *_ST_* and 99.9^th^/0.1^th^ percentile outliers for *Tajima’s D* are plotted in black per gene and labelled with gene symbols. **(B)** Genome wide landscape for Z chromosome. Ninety-fifth percentile outliers are plotted for *F* *_ST_* and 95^th^/5^th^ percentile outliers are plotted for *Tajima’s D* in grey, as calculated per gene. Ninety-ninth percentile outliers for *F* *_ST_* and 99^th^/1^st^ percentile outliers for *Tajima’s D* are plotted in black per gene and labelled with gene symbols.

Genome scans showed low genome-wide divergence between KP and WFP (**Figure 4**). The genome-wide *F* *_ST_* was 0.046, π of KP was 0.00262 and π of WFP was 0.00259, *d*_XY_ was 0.00306 with no *d* *_f_* (fixed difference) found. Twenty four thousand sixty-five blocks of 50kbp length were scanned, 22,148 of which were located on autosomes, 1,622 were located on Z chromosome and 295 unassigned. Since the W chromosome is very short (5.16Mb in chicken genome) and only males were used for population analyses, it was not included in the genomic landscape analysis. Examining autosomes and the Z chromosome separately, it was found that the average *F* *_ST_* of autosomal blocks was 0.043, the average *d*_XY_ was 0.00310, π of KP was 0.00268 and π of WFP was 0.00266. The top 1% outlier blocks on autosomes, which have the highest *F* *_ST_* (average 0.230, peak 0.605), were found to have much lower polymorphism, than when examined at the genome-wide scale (뜲π_KP_ = 0.0012, π_WFP_ = 0.0011, p < 0.001). The Z chromosome was more divergent than autosomes (average *F* *_ST_* = 0.089, average *d*_XY_ = 0.00247, π_KP_ = 0.0020 and π_WFP_ = 0.0019). The highest 1% outlier blocks on the Z chromosome had an average *F* *_ST_* of was 0.664, and the peak value was 0.741. The average π was 0.0003.

We identified genes which had high levels of divergence between species, by calculating population statistics per gene (**Figure 4**). Six hundred ninety-one autosomal genes had *F*_ST_ higher than 0.184 and 41 genes on the Z chromosome had *F* *_ST_* higher than 0.262 (**Supplementary Table 18**). GO enrichment analyses of autosomal genes with high *F* *_ST_* found that no categories were enriched for biological processes. Integral component of membrane was enriched for cellular components. No categories were enriched when looking at the genes with high *F* *_ST_* on the Z chromosome. A total of 339 autosomal genes were found with a *Tajima’s D* ≥ 1.565 (**Supplementary Table 19**) and 235 genes with *Tajima’s D* ≤ −1.179 for KP (**Supplementary Table 20**). No GO categories were enriched among genes with high or low Tajima’s D in KP. Three hundred twenty-five genes had values of *Tajima’s D* ≥ 1.669 (**Supplementary Table 21**) and 216 had values of *Tajima’s D* ≤ −1.191 for WFP (**Supplementary Table 22**). GO enrichment analysis revealed an overrepresentation of high *Tajima’s D* genes associated with microtubule cellular component categories and genes with low *Tajima’s D* had an overrepresentation of genes associated with proteolysis for biological function categories. For KP 18 genes on the Z chromosome had a *Tajima’s D* ≥ 1.723 and 12 genes had *Tajima’s D* ≤ −1.234. GO enrichment was not performed due to the small number of high diversity genes on the Z chromosome. WFP had 19 genes on the Z chromosome had *Tajima’s D* ≥ 1.835 and 10 genes had *Tajima’s D* ≤ -1.445. These results suggest species specific selection.

## Discussion

We have utilized methods of mitochondrial and nuclear whole genome *de novo* sequencing to shed unprecedented insight into the evolution and demographic histories of two small plover species. Although previous studies suggest KP and WFP are sufficiently differentiated at a phenotypic level to justify their classification as two different species (Kennerley et al., 2008; Rheindt et al., 2011), genetic studies have not always agreed (Rheindt et al., 2011). Our results show that KPs and WFPs have low levels of differentiation on average at the genomic level, with moderate to high differentiation in some regions. Our divergence time estimates suggest that Kentish and white-faced plovers diverged relatively recently, about 0.86 million years ago.

Using genome wide data, we were able to model complex demographic histories of the two plovers. ABC analysis estimates the divergence time to be about 863 thousand years ago during the Pleistocene. Estimates of Ancestral *N* *_e_* suggests that the most recent common ancestor had a high *N* *_e_* (0.75 million). KP was found to have a much larger effective population size than WFP. *N* *_e_* estimate for KP (3.58 million) were about 5.6 fold of WFP (0.64 million). *N* *_e_* can be an important estimate of the health of a population in terms of conservation biology. This can be particularly valuable for species where census data is lacking, such as with the WFP which has no census data available from the IUCN Red List, although the relationship between *N* *_e_* and census *N* can be difficult to interpret (Nadachowska-Brzyska et al., 2015).

We found contrasting demographic histories between the two plover species as demonstrated from the PSMC estimates, especially through their history from 1 million to approximately 100 thousand years ago. The *N* *_e_* of KP was about six-fold larger than WFP towards the end of the last glacial period (LGP) resulting from population increases in KP and declines in WFP. Although declines in population size at the start of the LGP is a common pattern in bird species (Nadachowska-Brzyska et al., 2015), marked contrasting patterns in population demography may reflect species differences in response to historical climate fluctuation and also population divergence (Groenen et al., 2012). One possibility is that a decrease in suitable habitat for WFP during the LGP could explain continued declines of the *N* *_e_* of WFP whereas the KP may have been able to exploit a wider range of habitats (Wang et al., 2019). Caution should be exercised when implementing demographic approaches such as ABC and PSMC and when interpreting the results, due to heavy dependence on the parameters and priors (Nadachowska-Brzyska et al., 2013; Nadachowska-Brzyska et al., 2016). For example, our estimation of *N* *_e_* had wide confidence intervals, probably due to the uncertainty generated from simulating population changes. Moreover, we applied constant *N* *_e_* and changing *N* *_e_* to estimate divergence patterns of the two plovers. Our model selection analysis showed that the estimate of *N* *_e_* indeed influenced the inference of the divergence scenario (**Supplementary Table S17**). Alternative analytical approaches, such as multiple sequentially Markovian coalescent analysis (MSMC) (Schiffels and Durbin, 2014) and SMC++ (Terhorst et al., 2017) can be employed to verify patterns and parameters when high-coverage sequencing data are available.

It is unknown whether the breeding grounds of KP and WFP overlap or whether any form of reproductive isolation has occurred. If a hybrid zone exists it has been estimated that this would be found in a narrow range in Fujian Province (Rheindt et al., 2011, Wang et al., 2019). It is believed that advanced reproductive isolation occurs relatively late during speciation events in birds, with complete F_1_ hybrid sterility often taking in the order of millions of years (Blundell et al., 2002; Nadachowska-Brzyska et al., 2013). The recent split of the two plover species would suggest that reproductive isolation may be limited. The rate of gene flow and recombination occurring throughout the genome can affect the rate at which reproductive isolation occurs. Migratory shorebirds are highly mobile with the potential to disperse large distances to breed, this can lead to high levels of gene flow and weak population structuring in some species (Küpper et al., 2012; Verkuil et al., 2012; Eberhart-Phillips et al., 2015; D’Urban Jackson et al., 2017). We estimated levels of gene flow throughout the history of the two species to determine whether historic gene flow occurred as a result of secondary contact, or alternatively, that gene flow has occurred continuously as the species diverged. Various scenarios of gene flow were modelled using ABC analysis. ABC simulations best fit the secondary contact model, in which the two plover species diverged allopatrically, with gene flow occurring after secondary contact. There was little support for models of isolation with migration, isolation, or early migration models. Extensive gene flow was found to be bidirectional but biased, with a higher level of immigration from WFP to KP. This might be comparable to the very large estimated *N* *_e_* of KP. It is expected that very few individuals are needed to exchange genes between populations to break down differentiation produced by genetic drift (Wright, 1931; Slatkin, 1987). How both phenotypic and genomic differentiation is maintained despite gene flow is a key question in evolutionary biology. Our results suggest that some form of selection must operate on these two species, especially on WFP, to maintain such divergence between the populations with the presence of historical gene flow (results from the present study) and concurrent gene flow (Wang et al., 2019). Further study of these species at the hybrid zone would help elucidate which selective forces might be maintaining these differences.

Taking advantage of whole genome resequencing, we detected highly differentiated genomic regions that might be involved in species divergence (Rogers and Bernatchez, 2007; Barrett and Hoekstra, 2011; Ellegren et al., 2012; Poelstra et al., 2014; Hoban et al., 2016). We used a window-based approach to perform whole genome scans and calculate various population statistics, including *F* *_ST_*, *d*_XY_, *π* and *Tajima’s D*, to characterize patterns of heterogeneity of differentiation across the two plovers’ genomes and to identify outlier loci. We found that average *F* *_ST_* across genome was 0.046. This is slightly higher than levels found between carrion and hooded crows, another taxonomically debated pair of species (Poelstra et al., 2014). It has been suggested that areas of peak differentiation contain genes involved in reproductive isolation and that these areas can contain genes responsible for differences in phenotype (Turner et al., 2005; Harr, 2006; Nosil et al., 2009; Ellegren et al., 2012; Poelstra et al., 2014). Higher levels of *F* *_ST_* and *Tajima’s D* in KP were found in the PPP3CB gene, which is part of the oocyte meiosis KEGG pathway and could therefore be important for promoting genetic incompatibility in females. Hybrid incompatibility often occurs in the heterogametic sex due to Haldane’s Rule (Haldane, 1922), which often means in birds that hybrid females are sterile and fertile males allow gene flow to occur between species (Mořkovský et al., 2018). However, it is unlikely that this single genetic region acts as a reproductive barrier as we did not find any fixed differences. Moreover, the genome-wide average *d*_XY_ was low (3.1×10^−3^) and high values of *d*_XY_ did not correlate with peaks of *F* *_ST_* (**Figure 4**). Therefore, it is not necessarily the case that this region has been involved in reducing introgression between the two plovers. An alternative interpretation of the high level of differentiating regions among closely related species is that these are due to local selective sweeps and background selection of regions with low recombination (Cruickshank and Hahn, 2014; Burri et al., 2015; Burri, 2017; Van Doren et al., 2017). In our study species, given the existence of gene flow, the few regions with elevated levels of differentiation suggests this may be driven by linked selection.

We observed a similar pattern on the Z chromosome (**Figure 4B**) as seen on the autosomes, with higher levels of block-average and peak relative divergence (0.089 and 0.741 respectively) and low average *d*_XY_. Particularly, a region with several genes (e.g. CLTA, PIGG, and CHRNB3) shows peak values of *F* *_ST_*, but relatively low average level of *d*_XY_. Because of the lower *N* *_e_* of the Z chromosome compared with the autosomes, this can result in faster lineage sorting and higher *F* *_ST_* (Wolf and Ellegren, 2017). Thus, the average relative divergence levels of sex chromosomes are often higher than on the autosomes (Ellegren et al., 2012; Poelstra et al., 2014). An overabundance of highly differentiated loci on the sex chromosomes are often detected (Payseur and Rieseberg, 2016). Several authors have proposed that selection is not necessarily the sole force contributing to divergence in genomic regions (Cruickshank and Hahn, 2014; Burri, 2017; Ravinet et al., 2017). It is possible that historic demographic fluctuations, such as population bottlenecks in WFP and recent expansion in KP, has also contributed to heterogeneity of genomic differentiation (Wolf and Ellegren, 2017). The force of selection operates more efficiently in populations with larger *N* *_e_*, while populations with a smaller *N* *_e_* are predominately subject to genetic drift. We therefore expect that the contrasting demographic histories of the two plovers have affected the patterns of genome heterogeneity, especially the Z chromosome that has a reduced *N* *_e_*.

This study also provides a greater understanding about the demographic histories of two shorebird species, especially linking their current population status with evolutionary context (Allendorf et al., 2001; Fitzpatrick et al., 2015; Fuentes-Pardo and Ruzzante, 2017). Although KP have a large census population size and are widely distributed throughout Eurasia and North Africa, there is evidence that populations are in decline in East Asia. The decline in Chinese plovers could, at least in part, be due to a reduction in suitable breeding and feeding locations along the Chinese coast due to land reclamation and development (Ma et al., 2014). Extremely low nest survival in Kentish plovers from Bohai Bay has been reported and linked to anthropogenic disturbance (Que et al., 2015). This work also emphasizes the lower effective population size of WFP compared to KP, which may have resulted from population declines occurring since the LGM (**Figure 3C** and Wang et al., 2019). Thus, increased effort to monitor population trends for this species is warranted in order to accurately assess any potential threats to this species and for conservation status evaluation for the IUCN Red List.

In conclusion, we produced the first high quality genome of the KP and performed whole genome resequencing of two plover species relatively early in their divergence. We found multiple pieces of evidence to support that the WFP and KP are distinct lineages with complex demographic histories. We also suggest scenario of gene flow between these two species due to secondary contact. Our results further reveal a heterogeneous pattern of genomic differentiation with elevated divergence in the Z chromosome. This suggests that some form of selection is working to maintain genetic and phenotypic differences between the two species. Overall, this study provides new insights into the genomic patterns between a species pair at an early stage of speciation. Further analyses of populations at the hybrid zone would increase our understanding of the specific selective forces maintaining this divergence.

## Data Availability Statement

Genomic sequences have been deposited at DDBJ/ENA/GenBank under the accession VUYV00000000. Other datasets contained in this manuscript will be made available by the authors on request.

## Author Contributions

YL and TS conceived of the study. XW and YL designed the study. PQ, QH, BW, ZZ and YL collected the samples. XW, NZ and KM, CZ, SL, BW, DC, and XY analyzed data. KM, AU and YL wrote the manuscript with contributions from all authors. All authors read and approved the final version of the manuscript.

## Funding

This work was supported by National Natural Science Foundation of China (31301875, 31572251 to YL, and 31600297 to PQ) and Special Program for Applied Research on Super Computation of the NSFC-Guangdong Joint Fund (the second phase) under Grant No. U1501501; a National Environment Research Council Great Western Four+ Doctoral Training Partnership studentship (grant number NE/L002434/1), Korner Travelling Award and a British Council and Chinese Scholarship Council Newton Fund PhD Placement awarded to KM; a Royal Society Dorothy Hodgkin Research Fellowship (grant number DH071902), Royal Society research grant (grant number RG0870644), a Royal Society research grant for fellows (grant number RG080272) and a NERC grant (NE/P004121/1) to AU. All sequencing data will be deposited in NCBI databases upon acceptance.

## Conflict of Interest

Author CZ and XY were employed by company Shenzhen Realomics Biological Technology Ltd. The remaining authors declare that the research was conducted in the absence of any commercial or financial relationships that could be construed as a potential conflict of interest.
